# A New Species of *Diploderma* (Squamata, Agamidae) from the Valley of Dadu River in Sichuan Province, with a Redescription of Topotypes of *D. splendidum* from Hubei Province, China †

**DOI:** 10.3390/ani14091344

**Published:** 2024-04-29

**Authors:** Bo Cai, Fengjing Liu, Dong Liang, Mian Hou, Huaming Zhou, Jiayun Zhong, Jing Li, Jiang Chang

**Affiliations:** 1 Chengdu Institute of Biology, Chinese Academy of Sciences, Chengdu 610299, China; caibo@cib.ac.cn; 2 College of Life Science, China West Normal University, Nanchong 637009, China; liufj106@163.com; 3 Sichuan Mt. Emei Forest Ecosystem National Observation and Research Station, Leshan 614201, China; emsstz@163.com; 4 College of Continuing (Online) Education, Sichuan Normal University, Chengdu 610066, China; turtlechina@126.com; 5 Ganzi Institute of Forestry Research, Kangding 626001, China; zhhm2@126.com (H.Z.); lygzzlj@sina.cn (J.L.); 6 Bureau of Ecology and Environment of Jinkouhe, Leshan 614700, China; 18981348259@163.com; 7 State Key Laboratory of Environmental Criteria and Risk Assessment, Chinese Research Academy of Environmental Sciences, Beijing 100012, China

**Keywords:** Eastern Tibetan Plateau, Hengduan Mountain Region, Xiling Gorge, Ichang City, dry valley

## Abstract

**Simple Summary:**

The genus *Diploderma* Hallowell, 1861 (Squamata, Agamidae), currently consists of 46 species, which are distributed across East Asia (inclusive of Japan) and the northern part of the Indochinese Peninsula, primarily inhabiting dry valley regions in Western China. This article introduces a novel species of *Diploderma* based on its unique morphological features and molecular evidence, discovered in the lower valley of the Dadu River in Sichuan Province, Western China. Phylogenetic analysis using *ND2* data suggests that this new taxon is distinct from its congeners. Morphologically, the new species can be differentiated from other *Diploderma* species by an assemblage of 46 specific characteristics. Principal component analysis (PCA) further demonstrates that this new species is clearly distinguishable from its closest relative, *D. splendidum*. Given these multiple lines of evidence, we describe this species from the lower Dadu River valley as a newly identified species, *D. daduense* sp. nov. This discovery brings the total number of recognized species within the genus *Diploderma* to 47.

**Abstract:**

This study describes a novel species of *Diploderma* (Squamata, Agamidae) from the lower valley of the Dadu River of the Sichuan Province of Western China based on its distinct morphological features and molecular evidence. *D. daduense* sp. nov. can be distinguished from its congeners by its tympanum concealed; head mainly green-yellow, supplemented by black; skin folds under the nuchal and dorsal crest obviously present in adult males only, its vertebral crest discontinuous between nuchal and dorsal sections with a distinct gap; transverse gular fold present but not obvious in some individuals; gular spot absent in both sexes; dorsolateral stripes green-yellow anteriorly, cyan in the center and blurry off-white posteriorly in adult males, the upper edge of dorsolateral stripes strongly jagged in adult males; no radial stripes around the eyes; inner-lip coloration smoky-white, and the coloration of the tongue and oral cavity as a light-flesh color in life; bright green-yellow transverse stripes on dorsal body in males; black patches are evenly distributed along the vertebral line between the dorsolateral stripes from the neck to the base of the tail in males; beech-brown or gray-brown line along the vertebral line with heart-shaped or diamond-shaped black patches on the dorsal body in females; and supratemporals fewer than four on at least one side. The phylogenetic tree based on mitochondrial *ND2* sequences indicates that *D. daduense* sp. nov. forms an independent clade with strong support 1/100 in ML bootstrap/Bayesian posterior probability and is the sister group to *D. splendidum*. At the inter-species level, the p-distance is at least 6.95%, further confirming that an independent species had been identified. Our work raises the number of species within the genus *Diploderma* to 47.

## 1. Introduction

*Diploderma* Hallowell, 1861, is one of the most diverse genera within the family Agamidae, comprising at least 46 recognized species, distributed throughout East Asia (including Japan) and the northern part of the Indochinese Peninsula [1,2]. Extensive field surveys and the application of DNA barcoding technology have significantly advanced our understanding of species diversity within the *Diploderma* genus.

In molecular systematics, these species form two distinct major clades on the phylogenetic tree [1]. One clade encompasses species distributed in the central or southern parts of the Hengduan Mountains, whereas the other clade primarily consists of species found in the eastern part of the Hengduan Mountains, extending their range to Taiwan Island and Japan. The minimum uncorrected pairwise distance among these species is significantly greater than the genetic distance between the two recognized species *D. drukdaypo* and *D. vela*, which stands at 2.6% [1].

Most *Diploderma* species exhibit prominent gular spots, and variations in the coloration of these spots play a crucial feature in species classification and identification [3]. Additionally, the color and pattern of dorsolateral stripes, as well as the presence or absence of radiating marks around the eyes, contribute to distinct traits for differentiation [4]. *Diploderma* species display typical sexual dimorphism in morphometric measurements, with males generally being more vividly colored than females [1,5].

The majority of *Diploderma* species are distributed along dry valley regions, particularly in the valleys of the Jinsha, Yalong, Nu, and Lancang rivers, displaying remarkable diversity and endemism across a wide altitudinal range [3]. Notably, numerous *Diploderma* species occupy and adapt to different stretches of river valleys within the Hengduan Mountains. In 2022, Shi et al. conducted a habitat preference and morphological differentiation study on nine *Diploderma* species along the Nu, Lancang, and Jinsha rivers, discovering that lizards from different habitat types exhibit specific preferences for habitat factors and have undergone relatively conspicuous differentiation in body size and locomotion characteristics between shrub-type and forest-type lizards [6].

**Figure 1:**
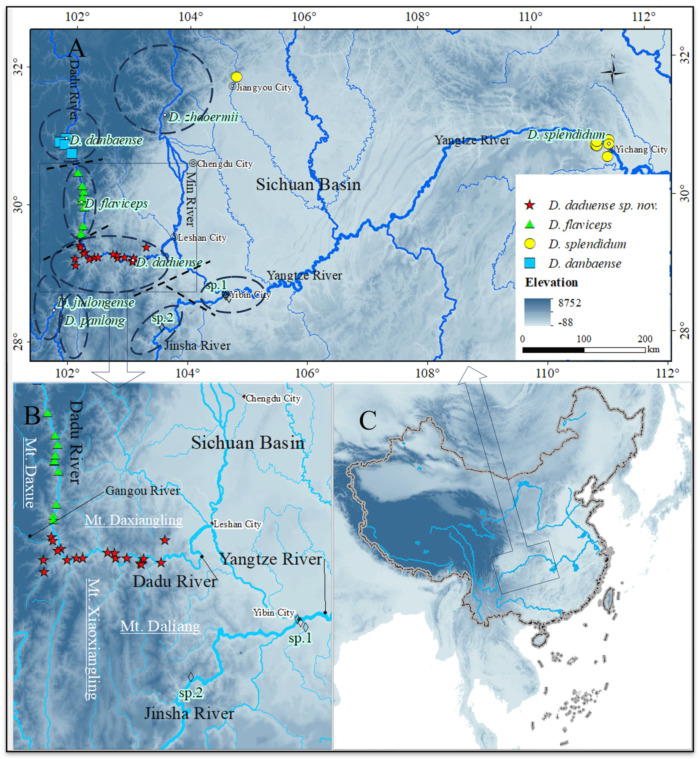
A map showing the locations of *Diploderma* specimens in the Dadu River Valley (**B**) and the locations of *D. splendidum* topotypes in Yichang (**A**,**C**). The red five-pointed star is *D. daduense* sp. nov., the green triangle is *D. flaviceps*, the yellow circle is *D. splendidum*, and the blue square is *D. danbaense*. The dotted coil represents the approximate distribution range of species in and around the Dadu Valley, and the dotted line represents the approximate distribution boundary of these species. The elevation data were obtained from Geospatial Data Cloud (2022).

However, the dry valleys of the Dadu River have received less attention. The Dadu River is a crucial tributary of the Yangtze River, joining the Min River at Leshan City before merging with the Jinsha River to form the Yangtze River at Yibin City. To date, three species of *Diploderma* (Figure 1) have been identified in the Dadu River Valley [1]: *D. danbaense* Liu, Hou, Ananjeva, and Rao, 2023 and *D. flaviceps* (Barbour & Dunn, 1919) above Luding County, while the area between Luding County and Leshan City is believed to harbor *D. splendidum* (Barbour & Dunn, 1919). *D. splendidum*’s distribution spans across the four provinces (Yunnan, Sichuan, Hubei, and Chongqing) [7], where there are significant variations in climate and terrain. The downstream reaches of the Dadu River are more than 700 km away from Yichang City, the type locality of *D. splendidum*, and the difference in climate conditions between these two areas is substantial. Populations of *D. splendidum* in these two regions also exhibit morphological differences.

In the period from 2018 to 2022, we conducted a series of field surveys in and around the Dadu River Valley and the gorge vicinity of Yichang City, during which we collected a series of specimens initially identified as *Diploderma splendidum*. Following molecular phylogenetic analyses and morphological comparisons, it became evident that the population from Dadu River Valley exceeds the threshold of intraspecific variation. Consequently, we describe one new species of *Diploderma* herein.

## 2. Materials and Methods

### 2.1. Sampling

Fieldwork was conducted in the vicinity of the Dadu River in Sichuan Province between June 2018 and June 2021 by Bo Cai, Yayong Wu, Ling Li, and Dong Liang and in the area surrounding Yichang City, Hubei Province, in August 2022 by Bo Cai. Live animals were photographed to document their color patterns prior to euthanasia. Euthanasia was performed through the intracelomic injection of 250 mg/kg of 1% MS-222 (Chengdu, China) for anesthesia, followed by intracelomic injection of 75% alcohol (Chengdu, China). Liver tissue samples were collected and stored in 95% ethanol for molecular analyses, and specimens were preserved in 75% ethanol.

Specimens were deposited at the Museum of Herpetology, Chengdu Institute of Biology, Chinese Academy of Sciences (CIB), the Museum of Yibin Key Laboratory of Animal Diversity and Ecological Conservation, Yibin University (YBU), and the Kunming Natural History Museum of Zoology, Kunming Institute of Zoology, Chinese Academy of Sciences (KIZ).

### 2.2. Morphological Analysis

Morphological data of recognized *Diploderma* species were obtained from the examination of museum specimens (Table S1), including CIB, YBU, and KIZ, and from the literature by Zhao et al. (1999) and Liu et al. (2023) [1,4].

Measurements were taken mainly following Zhao et al. (1999) [4] to the nearest 1 mm using a steel ruler for snout–vent length and tail length and to the nearest 0.1 mm using a digital caliper for other relatively short measurements. In total, 11 measurements were measured: the snout–vent length (SVL) from the snout tip to the anterior edge of the vent; tail length (TAL) from the anterior edge of the vent to the tip of the tail; head width (HW) between the widest points in the temporal region, anterior to the tympanum; head length (HL) from the tip of the snout to the right angle of the jaw; head depth (HD) at the temporal region of the head; snout–eye length (SEL) from the tip of the snout to the anterior margin of the orbit; length of the tallest nuchal crest (TNC) from the bottom to the tip of the tallest nuchal crest; foreleg length (FLL) from the axilla to the tip of finger IV, excluding the claw, measured with the limb straightened; hindleg length (HLL) from the groin to the tip of toe IV, excluding the claw, measured with the limb straightened; trunk length (TRL) from the armpit to the groin; and toe IV length (T4L) from the tip of toe IV to the base between toe III and IV, excluding the claw. To avoid the effects of allometry, measurement data from larvae and subadult individuals were excluded from morphological comparisons.

The definitions of morphological characteristics and the counting methods also mainly followed Zhao et al. (1999) and Liu et al. (2023) [1,4] as follows: the supralabial scale count (SL) from the rostral to the corner of the mouth; nasal–supralabial scale rows (NSL) between the first supralabial and the nasal; infralabials (ILs) from the mental to the corner of the mouth; ventrals (VNs) counted in a straight line along the medial axis between the transverse gular fold and the anterior edge of the cloaca; gulars (GUs) counted in a straight line along the medial axis between and excluding the mental and transverse gular fold; middorsal crest scales (MD) counted longitudinally from the first nuchal crest to the scale above the cloaca; finger IV subdigital lamellae (F4S) from the base between finger III and IV to the tip of finger IV, excluding the claw; toe IV subdigital lamellae (T4S) from the base between toe III and IV to the tip of toe IV, excluding the claw; radial stripes below the eyes (RSBE) absent or present; gular fold state (GF) absent or present; gular pouch state (GP) absent or present; gular spot color (GSC); tympanum state (TS) concealed with scales or not; skin fold under the nuchal crest (SFNC) absent or present; nuchal crest state (NC) strongly erected or not; skin fold under the dorsal crest (SFDC) absent or present; shape of dorsolateral stripes (SDS) in males, either smooth edged or jagged; ventral scale state (VSS) absent or present; hindlimbs adpressed forward (HAF) to the reaching area; and supratemporals (ST) enlarged, modified temporal scales.

Coloration descriptions are used terminology and codes in the RGB (red, green, blue) color model [8]. Data on coloration and ornamentation were collected from live specimens in both unstressed and stressed states, including the following: inner-lip coloration (ILC); the coloration of the oral cavity (CO): defined as the background coloration of the anterior roof and sides of the mouth, excluding the posterior palate and deep throat; the coloration of the tongue (CTG): defined as the coloration of the tongue; the coloration of the dorsolateral stripes (CDS): defined as the background coloration of the dorsolateral stripes; ventrolateral body coloration (VLBC); and ventral body coloration (VBC).

A principal component analysis (PCA) was utilized to assess whether newly collected specimens and species occupied distinct positions in morphospace and to confirm the consistency of the results with the species descriptions provided by molecular phylogenetic analyses. Characters used in the PCA were mensural data from SVL, TAL, HL, HW, HD, SEL, FLL, HLL, T4L, and TRL, with morphological data for *D. flaviceps* taken from Liu et al. 2023 [1]. The PCA was performed using the prcomp command in R version 4.3.2 [9] and scatterplotting was conducted with the R package ggplot2 3.4.4 [10]. Considering that sexual dimorphism is acknowledged in morphometric measurements for species within the genus *Diploderma* [1,5,8], mensural data for each sex were analyzed separately.

### 2.3. Molecular and Phylogenetic Analysis

Total genomic DNA for the newly collected specimens was extracted from liver tissues with a standard three-step phenol–chloroform extraction method [11] and sequenced by using published primers (Jap_70F: CCACCAAACAACTACACCTA, Jap_1559R: GGATTAATGCCCTCTGGATT) [5]. The PCR and sequencing methods followed Liu et al. (2023) [1].

The acquired nucleotide sequences were initially subjected to forward and reverse strand proofreading and editing using SeqManin 7.1.0.44 the DNAstar 7.1 software package (DNAStar Inc., Madison, WI, USA) [12]. There were 45 novel sequences of *ND2* obtained in this work, and a total of 92 *ND2* sequences of 45 *Diploderma* species were downloaded from GenBank for phylogenetic analyses. Corresponding sequences of *Pseudocalotes brevipes* (AF128502), *Bronchocela cristatella* (KR053114), and *Laodracon carsticola* (OR544068) were downloaded from GenBank and used as outgroups (Table 1).

Sequences were aligned in MEGA 11 [13] using ClustalW [14] with default settings. Phylogenetic analyses were conducted using Bayesian inference (BI) and maximum likelihood (ML) methods implemented in PhyloSuite v1.2.3 [15] and MEGA 11, respectively. Before the phylogenetic analyses, the best evolutionary model was conducted and chosen under the Bayesian inference criteria (BIC) using ModelFinder [16]. The GTR + I + G4 model was selected as the best model for the mitochondrial gene. In the BI analyses, two runs were performed simultaneously with four Markov chains. The chains were run for 10,000,000 generations and sampled every 1000 generations. The first 25% of the sampled trees were discarded as burn-in, and then the remaining trees were used to estimate Bayesian posterior probabilities (BPPs); nodes with BPP values of 0.95 and higher were considered well supported [17,18]. In the ML analyses, 1000 bootstrap pseudoreplicates via the ultrafast bootstrap (UFB) approximation algorithm were used to construct a final consensus tree; nodes with UFB values of 95% and above were considered significantly supported [19]. Finally, uncorrected genetic pairwise distances (*p*-distances) for *ND2* were calculated using default parameters in MEGA 11 [13].

## 3. Results

### 3.1. Phylogenetic Analyses

**Figure 2:**
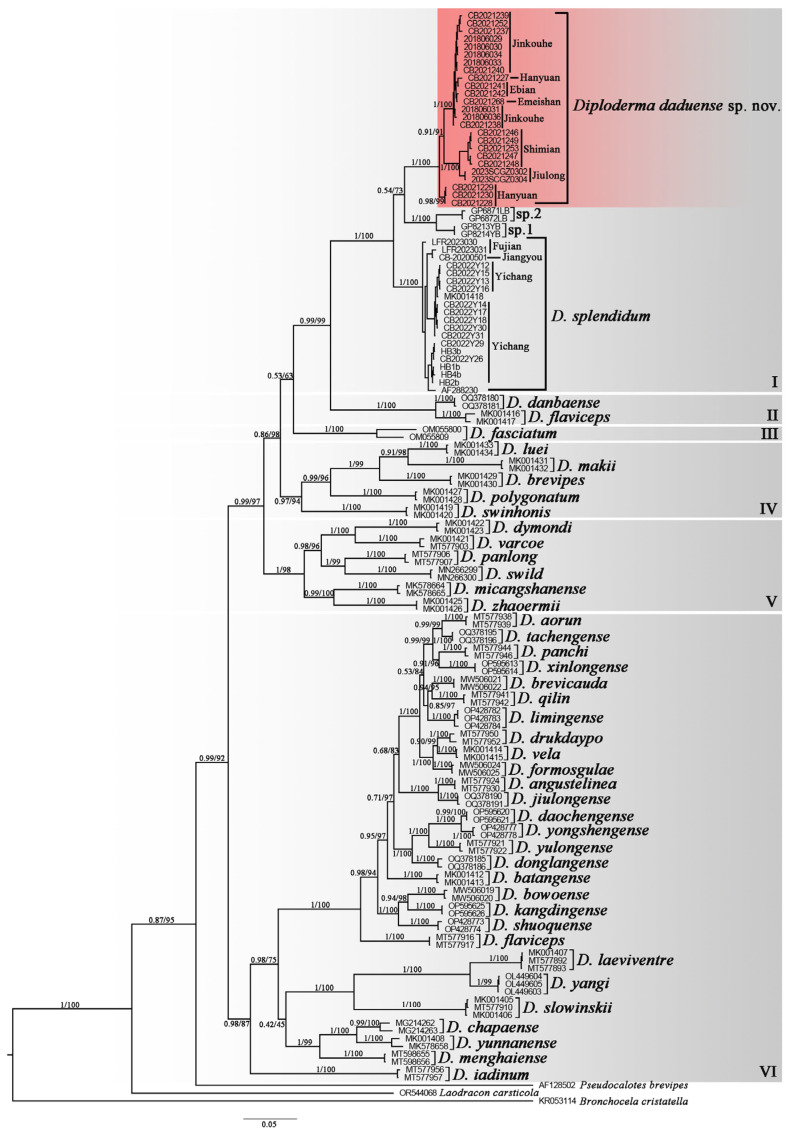
The phylogenetic tree of the genus *Diploderma* inferred from the mitochondrial *ND2* (974 bp). ML bootstrap support/Bayesian posterior probability support is denoted above each node. The maximum parsimony strict consensus tree for the *Diploderma* species is inferred from *ND2* gene fragments by using IQ-TREE v2.2.0 [20]; the *ND2* gene majority-rule consensus tree for the *Diploderma* species is inferred from partitioned Bayesian analyses by using MrBayes v3.2.7a [21]. In view of the unique clades of the Yibin and Leibo populations, sp.1 and sp.2 are used instead, respectively.

The *ND2* sequence alignments were 974 bp in length, and two consistent topologies of phylogenetic trees were obtained from BI and ML analyses, with all analyzed species of *Diploderma* forming a strongly supported monophyletic group. We further identified the presence of six unique subclades within this group. Among these, subclades I through V coalesced into a larger clade, which, in conjunction with the VI subclade, constituted the overall monophyletic group (Figure 2). The newly collected samples from the lower valley of the Dadu River population, Leibo population, Yibin population, and Yichang population are clustered into four distinct lineages in the context of subclade I, respectively, and are well supported. Specifically, clades from Yibin and Leibo clustered together first, then grouped with the population from the lower valley of the Dadu River, and finally aggregated with the population from the population from Yichang.

Uncorrected mean genetic distances ranged from approximately 1.44% to 22.69% across all samples, whereas the genetic distance between the population from the lower valley of the Dadu River population and other congeners is at least 6.95%, between the population from Yibin is 6.99%, and between the population from Leibo is 7.29%, which are much higher than the shortest genetic distance of the recognized *Diploderma* species (2.6% between *D. drukdaypo* and *D. vela*) (Table S2). However, the genetic distance between *D. daochengense* and *D. yongshengense* is only 1.44%, which may imply that one of these two species is a subspecies of the other, and we will conduct further research in the future.

### 3.2. Morphological Analyses

**Figure 3:**
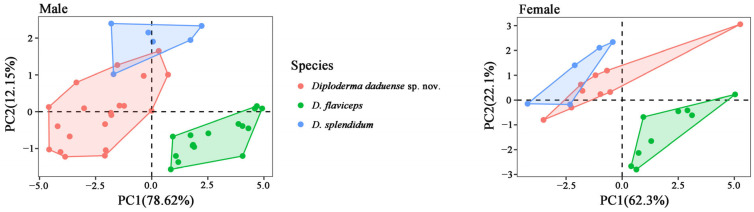
PCA based on ten morphometric characteristics (SVL, TAL, HL, HW, HD, SEL, FLL, HLL, T4L, and TRL) for *Diploderma daduense* sp. nov. (red), *D. flaviceps* (green), and *D. splendidum* (blue). Numbers inside the brackets indicate the percentages of the total variance explained by each axis.

From the results of the principal component analyses (PCA), it can be seen that the lower valley of the Dadu River population overlaps only slightly with the Yichang City population, and they are completely separated from *D. flaviceps* in morphospace (Figure 3). For the morphometric data of males, the cumulative proportions of the first two PCs accounted for 90.77% of the variance, and the eigenvalues were all greater than one. For the first two PCs, they load the most heavily on SVL, HL, HD, and HW (Table S3). For the female morphometric data, the cumulative proportion of the first two PCs accounted for 84.40% of the variance, and the eigenvalues were all greater than 1, as for males. For the first two PCs, they load the most heavily on HLL, SVL, HD, and HW (Table S4).

In addition, the population from the lower valley of the Dadu River also has its own unique morphological characteristics (Table 2). It showed different morphological characteristics from sister species *D. splendidum*: a bigger body size and heard in adult males, gular spot absence in both sexes; the upper edge of dorsolateral stripes is strongly jagged and lower edge smooth in adult males; and so on. We will elaborate on this in more detail in the following diagnosis.

### 3.3. Taxonomy Accounts

Both the molecular phylogenetic relationships and the uncorrected pairwise distance inferred from *ND2*, and the integration of the differences from morphological data and the morphospace, suggest that the lower valley of the Dadu River population represents one new species. We described it below.

#### 3.3.1. *Diploderma splendidum* (Barbour and Dunn, 1919)

*Japalura splendida* Barbour and Dunn, 1919 [22]

**Holotype**. USNM 35522, an adult male “from the gorge of the Yangtze River near Ichang (=Yichang City), Hupeh (=Hubei Province), central China”, collected by E. Blackwelder. After reviewing the original documents and the old map involved, we believe that this gorge should be the Lower Gorge section of Xiling Gorge in Yiling District, Yichang City, China.

**Topotypes**. All these topotypes were collected from Yichang City, Hubei Province, China, by Bo Cai in August 2022: One subadult male CIB-CB2022Y30, two females YBU23124(HB1) and YBU-HB3, four males YBU23125(HB2), YBU-HB4, CIB-CB2022Y29, and CIB-CB2022Y26 from Xiling Gorge Scenic Area, Yiling District; CIB-CB2022Y16 (subadult male) from Mt. Wenfo, which is south of Xiling Gorge, Dianjun District; CIB-CB2022Y15 (subadult male), CIB-CB2022Y14 (female), CIB-CB2022Y12 (female) and KIZ062362 (CB2022Y13, male) from Sandouping Town near the Three Gorges Dam, Yiling District; KIZ062363 (CB2022Y18, subadult female) and CIB-CB2022Y17 (male) from Songjia River, which is north of Xiling Gorge, Yiling District.

**Description of topotypes**. (1) Body size relatively large, SVL 69.7–83.3 (average 77.1) mm in adult males, 63.1–78.26 (average 70.1) mm in adult females; (2) tail relatively long, TAL/SVL 2.26–2.70 (average 2.47) in adult males, 1.32–2.67 (average 2.14) in adult females; (3) limbs moderately long, FLL/SVL 0.47–0.54 (average 0.50) in adult males, 0.45–0.52 (average 0.49) in adult females, HLL/SVL 0.78–0.89 (average 0.83) in adult males, 0.77–0.81 (average 0.79) in adult females; (4) head relatively robust, HW/HL 0.59–0.73 (average 0.65) in adult males, 0.53–0.65 (average 0.59) in adult females; (5) finger IV subdigital lamellae 18–23 (average 19.9), toe IV subdigital lamellae 21–31 (average 26.0); (6) tympanum concealed; (7) skin folds under nuchal and dorsal crest present in males only, vertebral crest continuous between nuchal and dorsal sections; (8) transverse gular fold present but not obvious in some individuals; (9) dorsolateral stripes smooth-edged in adult males, but capsule-shaped in adult females, subadult males, and Juveniles; (10) top of head covered with medium-sized, subequal, and rugose scales; (11) upper labials 7–9; (12) orbit separated from upper labials by 2–4 rows of scales, the middle row much enlarged; (13) lower labials 7–10; (14) a faintly indicated fold anterior to the insertion of the forelimb; (15) a large area above insertion of forelimb covered with tiny almost granular scales; (16) back and sides covered with imbricate, heterogeneous, and strongly keeled scales; (17) ventral scales of head almost homogeneous in size and keeled, with few slightly larger scales; (18) ventral scales of body homogeneous in size and strongly keeled; (19) scales of fore- and hindlimbs strongly keeled and heterogeneous in size; (20) fourth toe with claw crossing eye or reaching eye when hindlimbs adpressed forward; (21) supratemporals of both sides are greater than or equal to 4. These features of adults are consistent with the original description [22].

**Figure 4:**
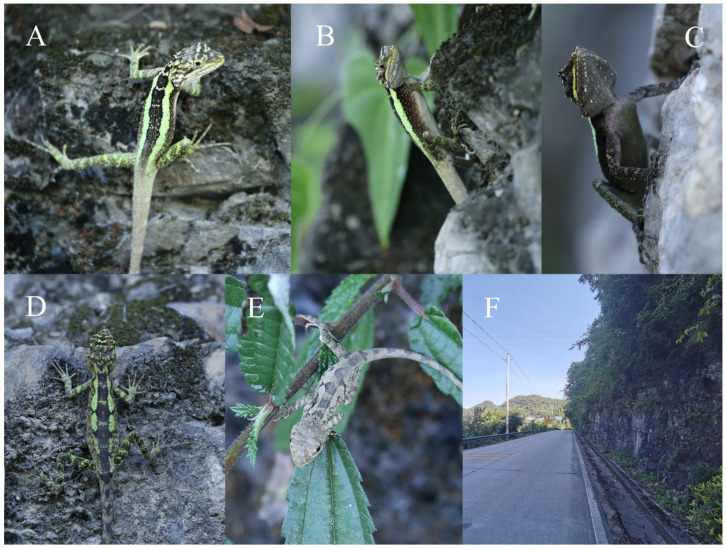
Topotypes of *Diploderma splendidum* in life. CB2021Y29 (male) dorsal view (**A**), lateral view (**B**), and ventral view (**C**); CB2021Y12 (female) dorsal view (**D**); CB2021Y44 (Juveniles) dorsal view (**E**); habitats of new species (**F**).

**Figure 5:**
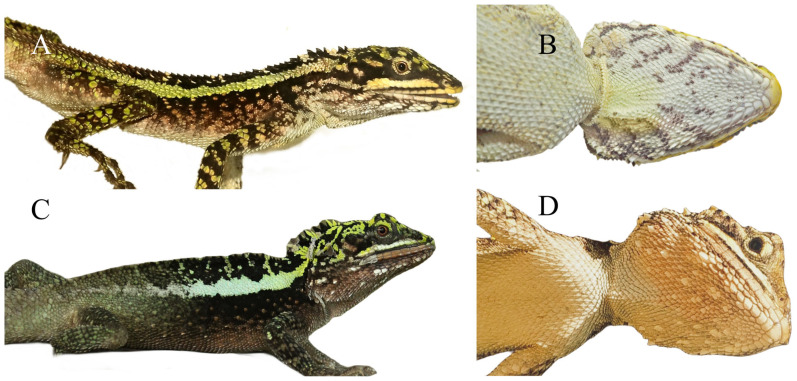
Comparison of males of two species in life. *Diploderma splendidum* CB2021Y29 lateral view (**A**), ventral view (**B**); *D*. *daduense* sp. nov. CIB119354 lateral view (**C**), ventral view (**D**).

**Coloration of topotypes in life** (Figure 4 and Figure 5A,B). In life, adult males have a yellow (255, 255, 0) gular spot, which is consistent with Barbour and Dunn (1919) [22] and Xu et al. (2023) [23], green-yellow to cyan (0, 255, 255) dorsolateral stripes, and adult females have green-yellow (173, 255, 47) dorsolateral stripes. No radial stripes around the eyes; the inner-lip coloration is smoky-white (245, 245, 245), and the coloration of the tongue and oral cavity is a light-flesh color (239, 205, 197) in life; green-yellow transverse stripes on the dorsal body, near-square-shaped black patches are evenly distributed along the vertebral line between the dorsolateral stripes from the neck to the base of the tail in both sexes. When stressed, the color of ventral head turns dim-gray (105, 105, 105) with slightly larger scales turning white (255, 255, 255), the ventral body coloration turning dim-gray, and the ventrolateral body turning black (0, 0, 0).

**Distribution and natural history of topotypes**. According to the nearby tributaries of the Yangtze River and the topography, the Xiling Gorge is divided into four sections from upstream to downstream: the Xiangxi Wide Canyon, Upper Wide Canyon, Miaonan Wide Canyon, and the Lower Gorge. Our sampling covers almost all of Xiling Gorge, which is near the Yangtze River, elevation 99–669 m. This population of *Diploderma splendidum* is terrestrial, inhabiting the central subtropical evergreen broad-leaved forest of the Xiling Gorge and its surrounding hills (Figure 1).

These lizards are commonly found on crevice-filled cliffs and on rock piles or soil walls at forest edges (Figure 4). All specimens were collected between 08:00 and 17:00 h in August.

*Diploderma splendidum* is oviparous. Specimens from Yichang City obtained in August had 2–6 eggs.

#### 3.3.2. *Diploderma daduense* sp. nov. Cai, Liu, and Chang

urn:lsid:zoobank.org:act:2ABDED00-9214-4FA1-8C8D-D4E571C5C561

*Japalura splendida* Barbour and Dunn, 1919 in part [22].

*Diploderma splendidum* Wang et al., 2019 in part [5].

**Holotype**. Adult male, CIB119354 (filed number CB2021251), collected from Jinkou Gorge (29.2292° N, 103.0568° E; at elevation 634 m a.s.l.), Leshan City, Sichuan Province, China, by Bo Cai in June 2021.

**Allotype**. Adult female, YBU-GP9889 (filed number CB2021252), the same collecting information as the holotype.

**Paratypes**. All these specimens are collected from Sichuan Province, China. Shimian County: one subadult female (CIB-CB2021253 from Liudaping (29.2629° N, 102.4148° E; at elevation 1181 m a.s.l.) and one subadult female (CIB-CB2021249), two subadult males (CIB-CB2021246–47) and one adult female (CIB-CB2021248) from Hongqi Village (29.2629° N, 102.4148° E; at elevation 877 m a.s.l.) collected by Bo Cai in June 2021; one subadult female (YBU22692) from Zhengjiaping (29.5055° N, 102.1764° E; at elevation 1040 m a.s.l.) and one adult male (YBU23205) from Longtoushi (29.3391° N, 102.2591° E; at elevation 1165 m a.s.l.), one adult male (YBU23209) from Xiaping Village (29.2401° N, 102.3279° E; at elevation 995 m a.s.l.), four adult males (YBU23202, YBU22688, YBU23210, and YBU23212) from Mafu Village (29.2636° N, 102.4803° E; at elevation 824m a.s.l.) collected by Ling Li and Yayong Wu in June 2022. Jiulong County: two adult males (CIB-2023SCGZ0301–02) from Wanba Town (29.1375° N, 102.1004° E; at elevation 1687 m a.s.l.) and two adult females (CIB-2023SCGZ0303–04) from Hongba Town (29.238° N, 102.0876° E; at elevation 2063 m a.s.l.) collected by Huangming Zhou and Jing Li in August, 2023. Hanyuan County: two adult females (CIB-CB2021228–29), one adult male (CIB-CB2021230), and one subadult female (CIB-CB2021227) from Sanguzhuang (29.2685° N, 102.8101° E; at elevation 880 m a.s.l.) collected by Bo Cai in June 2021; one adult male (YBU23204) from Zhaohoumiao (29.3087° N, 102.7964° E; at elevation 1053 m a.s.l.), one adult male (YBU22711) from Shunhe Township (29.3154° N, 102.7262° E; at elevation 842 m a.s.l.) collected by Ling Li and Yayong Wu in June 2022. Ganluo County: one adult female (YBU22706) and two adult males (YBU23208 and YBU23218) from Tianping Village (29.2731° N, 102.921° E; at elevation 857 m a.s.l.) collected by Ling Li and Yayong Wu in June 2022. Jinkouhe District: two adult males (CIB-CB2021237 and YBU22698), one subadult male (CIB-CB2021239), and two adult females (CIB-CB2021238 and CIB-CB2021240), same information as holotype; four adult males (CIB-201806030–33) and two adult females (CIB-201806026 and CIB-201806035) collected from Jinkou Gorge (29.2195° N, 103.0645° E; at elevation 639 m a.s.l.) by Bo Cai in June 2018. Ebian County: one male (CIB-CB2021242), two subadult males (CIB-CB2021241 and CIB-CB2021243) from Beifeng Mt. (29.2414° N, 103.261° E; at elevation 587 m a.s.l.) collected by Bo Cai in June 2021. Emeishan City: two subadult females (CIB-CB2021244–45) from Kuhaoping (29.4318° N, 103.2906° E; at elevation 1137 m a.s.l.) collected by Bo Cai in June 2021.

**Etymology**. The Latin specific epithet *daduense* is derived from the Dadu River, where the new species was discovered. The genus *Diploderma* is Greek neuter, and that *-ense* of this new species name is the Latin neuter combinatorial suffix denoting “pertaining to” or “originating in”. And we suggest Dadu Mountain Lizard as its English common name and 大渡攀蜥 (Chinese phonetic alphabet: dà dù pān xī) as its Chinese common name.

**Diagnosis**. *Diploderma daduense* sp. nov. can be diagnosed from other *Diploderma* species by the following unique combination of characters: (1) body size large, SVL 74.7–95.0 (average 86.5) mm in adult males, 52.6–80.2 (average 70.5) mm in adult females; (2) head relatively larger, HW/HL 0.61–0.79 (average 0.80) in adult males, 0.60–0.67 (average 0.64) in adult females; (3) finger IV subdigital lamellae 17–23 (average 19.2), toe IV subdigital lamellae 21–31 (average 25.3); (4) tympanum concealed; (5) head mainly green-yellow, supplemented by black; (6) skin folds under nuchal and dorsal crest obviously present in adult males only, vertebral crest discontinuous between nuchal and dorsal sections with a distinct gap; (7) transverse gular fold present but not obvious in some individuals; (8) ventral scales of body homogeneous in size and strongly keeled; (9) gular spot absent in both sexes; (10) dorsolateral stripes green-yellow anteriorly, cyan in center and blurry off-white posteriorly (248, 240, 227) in adult males, upper edge of dorsolateral stripes strongly jagged in adult males; (11) no radial stripes around eyes; (12) inner-lip coloration smoky-white, and coloration of tongue and oral cavity light-flesh color in life; (13) ventrolateral body coloration smoky-white, dim-gray, or rust-red (214, 107, 79); (14) bright green-yellow transverse stripes on dorsal body in males; (15) black patches are evenly distributed along the vertebral line between the dorsolateral stripes from the neck to the base of the tail in males; (16) beech-brown (87, 65, 40) or gray-brown (127, 112, 83) line along the vertebral line with heart-shaped or diamond-shaped black patches on dorsal body in females; and (17) supratemporals fewer than four on at least one side.

**Description of Holotype**. Adult male, body large-sized, SVL 93.5 mm; tail long, TAL 245.2 mm; head longer (30.0 mm) than wide (22.6 mm); head depth 15.1 mm; length of tallest nuchal crest 2.5 mm; snout–eye length 11.1 mm; foreleg length 42.4 mm; hindleg length 72.0 mm; toe IV length 18.3 mm; trunk length 49.0 mm; snout moderately long, SEL/HL 0.37. Rostral flat, bordered by 5 small postrostral scales; dorsal head scales heterogeneous in size, most of them keeled. Number of scales between eyes 17; nasal scale approximately oval; internasals 7; loreals 6/6, small, unkeeled; supralabials 8/8, infralabial scales 8/9, supratemporals 4/3, and middorsal crest scales 45, keeled; gular scales 33 and ventral scales 64; finger IV subdigital lamellae 21/23; toe IV subdigital lamellae 29/31; nasal–supralabial scale rows 1/1; suborbital scale rows 2/2. Below subocular, there are 3 parallel rows of scales, and first row extends from front of anterior nasal to upper post-rictus, second row extends from postrostrals to upper post-rictus, and third row is supralabials; transverse gular fold present; gular pouch present in life; tympanum concealed, covered with small keeled scales; well-developed skin fold under nuchal crest present, vertebral crest discontinuous between nuchal and dorsal sections with gap; dorsal and ventral scales distinctively keeled exclude scales around eyes and lips; dorsal scales of head, trunk, limbs, and tail heterogeneous in size, ventral scales of head homogeneous in size with few larger scales, ventral scales of trunk homogeneous in size; fold present in front of shoulder; fourth toe with claw reaching between tympanum and eye when hindlimbs adpressed forward. Upper edges of dorsolateral stripes are strongly jagged, but lower edge is relatively smooth.

**Figure 6:**
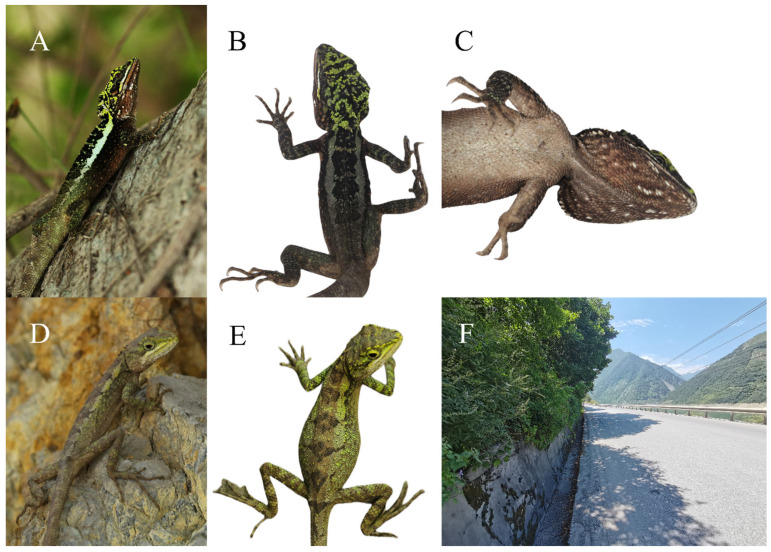
Types of *Diploderma daduense* sp. nov. in life. Holotype CIB119354 (male) lateral view (**A**), dorsal view (**B**), and ventral view (**C**); allotype YBU−GP9889 (female) lateral view (**D**), dorsal view (**E**); habitats of new species (**F**).

**Coloration of Holotype in life** (Figure 5C,D and Figure 6). The dorsal head is mainly green-yellow, with two black transverse stripes connecting the left and right supraoculars, black spots on the frontal, and black and green-yellow markings on the anterodorsal edge. The green-yellow ground color of the head is maculated with black. There are four stripes around the upper eye except the subocular regions on each side, with a thick black stripe extending from the posterior nasal through the lower palpebrals to the temporal region. The first subocular scale row is white in the middle and green-yellow at both ends, second row black. The supralabials and infralabials are white, turning gray-brown when stressed. Below are infralabials with beech-brown lines. Gular spot is absent. The ventral scales of the head are white with gray-brown lines, turning gray-brown with beech-brown lines and scattered with white spots when stressed. The inner-lip coloration is smoky-white, and the coloration of the tongue and oral cavity is a light-flesh color.

Five dorsal patches are black with small green-yellow spots; dorsolateral stripes are green-yellow anteriorly, cyan in the center, and blurry off-white posteriorly; the upper edge of dorsolateral stripes is strongly jagged, and the lower edge is relatively smooth. The ventrolateral body is light-gray (211, 211, 211), gray-brown to black with white or gray-brown spots; the venter is white but off-white to light-gray or gray-brown when stressed; the ventral sides of the limbs are the same as the venter. The dorsal sides of the limbs are gray-brown, interspersed with green (0, 128, 0) and black. The green transverse patterns of the forelimbs are clear, and those of the hindlimbs are blurred. The tail has 15 gray rings.

**Variation**. *Diploderma daduense* sp. nov. is sexually dimorphic: (1) males are larger than females, SVL 74.7–95.0 (average 86.5) mm vs. 52.6–80.0 (average 70.5) mm; (2) tail relatively long, TAL/SVL 2.04–2.62 (average 2.34) vs. 1.89–2.42 (average 2.22); (3) head relatively larger, HW/HL 0.61–0.79 (average 0.80) vs. 0.60–0.67 (average 0.64); (4) length of tallest nuchal crest longer, TNC 1.2–2.9 (average 1.9) mm vs. 0.5–1.4 (average 1.03) mm; (5) skin folds under nuchal and dorsal crest obviously present in adult males only; (6) dorsolateral stripes prominent present in adult males, absent or not noticeable in females; (7) black patches evenly distributed along vertebral line in males, heart-shaped or diamond-shaped black patches in most females.

**Comparisons**. *Diploderma daduense* sp. nov. differs from the topotypes of *D. splendidum* in Yichang City by having the following combined characteristics: (1) body size larger SVL 74.7–95.0 (86.5) mm vs. 69.7–83.3 (77.1) mm in adult males; (2) tail relatively shorter TAL/SVL 2.04–2.62 (2.34) vs. 2.26–2.70 (2.47) in adult males; (3) head depth larger HD/HL 0.46–0.54 (0.49) vs. 0.42–0.47 (0.44) in adult females; (4) snout–eye length shorter SEL/HL 0.36–0.47 (0.41) vs. 0.40–0.51 (0.43) in adult females; (5) foreleg length shorter FLL/SVL 0.41–0.49 (0.45) vs. 0.47–0.54 (0.50) in adult males, and 0.38–0.49 (0.44) vs. 0.45–0.52 (0.49) in adult females; (6) hindleg length shorter HLL/SVL 0.67–0.81(0.74) vs. 0.78–0.89 (0.83) in adult males; (7) length of tallest nuchal crest longer TNC/HL 0.04–0.09 (0.06) vs. 0.04–0.07 (0.05) in adult males; (8) gular spot absent in both sexes vs. yellow in adult males; (9) upper edge of dorsolateral stripes strongly jagged but lower edge smooth in adult males vs. both upper and lower edges smooth in adult males or capsule-shaped in subadult males; (10) vertebral line black patches heart-shaped or diamond-shaped in females vs. nearly square in females; and (11) supratemporals fewer than 4 on at least one side vs. 4–5.

In the absence of live color comparisons, this new species can be differentiated from the Yibin and the Leibo populations of *Diploderma splendidum* (sp.1 and sp.2) based on its unique combination of measurements traits. *D. daduense* sp. nov. compared to the Yibin population (sp.1) is as follows: (1) body size larger, SVL 74.7–95.0 (86.5) mm vs. 63.7–94.3 (79.2) mm in adult males, (2) tail relatively shorter TAL/SVL 2.04–2.62 (2.34) vs. 2.35–2.64 (2.55) in adult males, and 1.89–2.42 (2.22) vs. 2.32–2.63 (2.50) in adult females; (3) head width wider HW/HL 0.61–0.79 (0.80) vs. 0.60–0.69 (0.65) in adult males; (4) snout–eye length shorter SEL/HL 0.36–0.44 (0.39) vs. 0.41–0.46 (0.44) in adult males; and (5) hindleg length shorter HLL/SVL 0.67–0.81 (0.74) vs. 0.70–0.87 (0.78) in adult males. *D. daduense* sp. nov. compared to the Leibo population (sp.2) is as follows: (1) head depth smaller HD/HL 0.42–0.60 (0.49) mm vs. 0.46–0.71 (0.52) in adult males, (2) snout–eye length shorter SEL/HL 0.36–0.44 (0.39) vs. 0.41–0.63 (0.47) in adult males, and 0.36–0.47 (0.41) vs. 0.45–0.47 (0.46) in adult females; (3) trunk length longer TRL/SVL 0.43–0.52 (0.47) vs. 0.41–0.49 (0.45) in adult males, and 0.45–0.54 (0.49) vs. 0.43–0.49 (0.46) in adult females; and (4) head length HL/SVL shorter 0.27–0.30 (0.29) vs. 0.30–0.32 (0.31) in adult females.

The morphological differences between *Diploderma daduense* sp. nov. and *D. flaviceps* [1], which share a distribution border, are as follows: (1) body size larger SVL 74.7–95.0 vs. 60.5–75.6 in adult males, and 52.6–80.2 vs. 53.4–67.1 in adult females; (2) tail relatively longer TAL/SVL 2.04–2.62 vs. 1.88–2.09 in adult males; (3) dorsolateral stripes green-yellow, cyan to off-white (vs. pure yellow) in adult males.

*Diploderma daduense* sp. nov. differs from *D. dymondi*, *D. panlong*, *D. slowinskii*, *D. varcoae*, and *D. swild* in having concealed tympana (vs. exposed).

*Diploderma daduense* sp. nov. differs from *D. angustelinea*, *D. aorun*, *D. batangense*, *D. bowoense*, *D. brevicauda*, *D. chapaense*, *D. daochengense*, *D. donglangense*, *D. flavilabre*, *D. formosgulae*, *D. iadinum*, *D. jiulongense*, *D. kangdingense*, *D. laeviventre*, *D. limingense*, *D. menghaiense*, *D. panchi*, *D. qilin*, *D. tachengense*, *D. xinlongense*, *D. yangi*, *D. yongshengense*, *D. yulongense*, and *D. zhaoermii* by the absence of a gular spot in adult males in life (vs. the presence of a colorful gular spot).

*Diploderma daduense* sp. nov. differs from *D. drukdaypo*, *D. shuoquense*, *D. vela*, *D. swinhonis*, and *D. micangshanense* in the absence (vs. presence) of distinct radial stripes around the eyes.

*Diploderma daduense* sp. nov. differs from *D. yunnanense* by having indistinct oral coloration in life (light-flesh vs. light-chrome-orange).

*Diploderma daduense* sp. nov. differs from *D. brevipes*, *D. danbaense*, *D. hamptoni*, *D. makii*, *D. fasciatum*, *D. grahami*, and *D. szechwanensis* in adult males having green-yellow, cyan to off-white dorsolateral stripes vs. pure white, yellow, or absent dorsolateral stripes.

*Diploderma daduense* sp. nov. differs from *D. luei*, *D. ngoclinense*, and *D. polygonatum* by the presence of a transverse gular fold (vs. absence).

**Distribution and Natural History**. *Diploderma daduense* sp. nov. is terrestrial, inhabiting the central subtropical evergreen broad-leaved forest of the lower valley of the Dadu River and its surrounding hills (Figure 1), which is distributed in Shimian County, Jiulong County, Hanyuan County, Ganluo County, Ebian County, Jinkouhe District, Emeishan City, and other places in Sichuan Province, China, with the elevation 574–2063 m.

Compared with the warm–dry habitat of *Diploderma flaviceps* and *D. danbaense* in the upper reaches of the Dadu River, part of the habitat of *D. daduense* sp. nov is located in the West China Rain Screen Area, which is wetter and cooler, and the Fraction Vegetation Coverage is higher. Only near Hanyuan County in the central part of the distribution of *D. daduense*, the habitat is a warm–dry valley. *D. daduense* sp. nov is commonly found on tree branches, shrublands, crevice-filled rock piles, or soil walls at forest edges (Figure 6). All specimens were collected between 09:00 and 18:00 h in June and July.

*Diploderma daduense* sp. nov is oviparous, mating in April to May every year, and laying eggs in June to July [24]. We found this species to contain 2–7 eggs. The smallest female CIB-201806035 is only 52.6 mm in snout–vent length and has already conceived 2 eggs. Gao et al. (2004) studied the hatching of 6 eggs (12.6–13.8 [average 13.10] mm long and 8.0–9.0 [average 8.56] mm wide), found that the incubation period lasted approximately 49 days, from 20 June 2001 to 7 August 2001, and the snout–vent length of the four hatchlings was 23–25 (24.5) mm, and the tail length was 49–52 (51.3) mm [24]. We found this species to prey on larvae and adults of arthropods such as insects and spiders.

## 4. Discussion

The genus *Diploderma* boasts significant diversity, yet many of its species are characterized with insufficiently detailed distribution records and limited genetic data derived from multiple specimens. The discovery of *D. daduense* sp. nov. has notably augmented our comprehension of species within the genus *Diploderma*, providing a more comprehensive perspective on morphological variations, distribution limits, and genetic diversity. *D. daduense* sp. nov. exemplifies a new crucial role played by the Hengduan Mountains in the process of speciation. This finding not only aids in the investigation of the evolutionary relationships between the middle and upper reaches of the Yangtze River but also sheds light on the geological history of the Dadu River Valley. Moreover, it serves as a cornerstone for future studies in species ecology and related fields.

In genetics, sequences of *Diploderma daduense* sp. nov. have been resolved into three clades. These three clades correspond to geographical areas within the lower reaches of Luding County: The upper section, encompassing both Shimian and Jiulong, forms one clade; the middle segment around Hanyuan constitutes a separate clade; and the lower segment, extending from parts of Hanyuan downwards to Emeishan and Jinkouhe, makes up the third clade. Of note, among these specimens, there is one from Hanyuan that has been identified as belonging to the Jinkouhe clade. In contrast to the tight association between genetic information and distribution in *D. daduense* sp. nov., such a relationship is more dispersed in *D. splendidum*. The molecular sequence of the specimen from Jiangyou City and Fujian Province grouped with that of the topotypical *D. splendidum* specimens led Xu et al. (2023) to hypothesize that the Fujian population may be an introduced species resulting from wildlife trade and Buddhist release activities [23], implying that the Jiangyou population could have also been sourced in this manner. However, definitive confirmation necessitates the collection of additional samples and the implementation of microsatellite analysis. In contrast, *D. daduense* sp. nov. is currently minimally affected by the pet trade.

In the PCA, there are significant differences between the *Diploderma splendidum* group and *D. flaviceps*, while the morphospace for *D. splendidum* from Yichang also exhibited considerable divergence compared to those from *D. daduense* sp. nov. Unfortunately, we lack individual morphological data for sp.1 and sp.2, which means that their relationships in the PCA context with respect to *D. daduense* sp. nov. remain to be further investigated.

In climatological and geographical terms, the unique terrain of the Hengduan Mountains has given rise to a distinct climatic–geographical unit known as dry valleys. These valleys exhibit higher temperatures and lower humidity compared to their surrounding areas. Based on differences in temperature and humidity, the arid valleys in southwestern China can be classified into three categories: hot–dry valleys, warm–dry valleys, and temperate–dry valleys [25]. The local climatic–geographical units along the Dadu River are notably associated with species from *Diploderma* [25,26,27,28]. The distribution area for *D. danbaense* is primarily situated in “semi-arid” warm–dry valleys near Danba, while *D. flaviceps* inhabits “semi-arid but slightly more humid” warm–dry valleys close to Luding. The habitat range for *D. daduense* sp. nov. extends across “semi-arid but humid” warm–dry valleys and downstream into the West China Rain Zone. The species sp.1 in Leibo is native to the hot–dry valley of the Jinsha River. The city of Yibin where sp.2 is found belongs to a subtropical monsoon climate zone with moderate moisture, which also exhibits characteristics of a South Asian tropical climate in its low hills and valleys [29]. The Yiling District, where the type locality of *D. splendidum* is located, sits within a mid-subtropical monsoon climate region featuring distinct seasons, mild climates, and moderate precipitation [30]. These instances appear to demonstrate that different geographical and climatic environments give rise to distinct species.

In the species distribution boundaries, *Diploderma daduense* sp. nov., similarly to other *Diploderma* species within the Hengduan Mountains, is characterized as a valley-dwelling species. The valley-type distribution of *D. daduense* is bounded to the west by Mount Daxue, to the south by Mount Xiaoxiangling and Mount Daliang, to the north by Mount Daxiangling, and to the east by the Sichuan Basin. The upper portion of the valley-type distribution for *D. daduense* directly borders, yet does not overlap with, the lower reaches for *D. flaviceps*, where their western distribution boundary is demarcated by the Dagou River at the confluence of Luding and Shimian counties, while the eastern distribution boundary is estimated be situated about 20 km away from the Dagou River mouth, based on existing specimens. To date, no evidence of overlapping distributions has been found between *D. daduense* and *D. flaviceps* nor have any hybrid individuals been morphologically identified. The Yangtze River potentially acts as a separator between *D. daduense* and sp.1 found in Yibin, which is currently known only from the southern bank of the Yangtze River in Yibin City. Moreover, Mount Daliang serves as a barrier preventing contact between *D. daduense* and sp.2 found in Leibo County. As a result, geographically, *D. daduense* is isolated from *D. flaviceps* and other members of the *D. splendidum* species group, with no currently documented instances of overlapping distribution areas.

As a major tributary of the Yangtze River, the Dadu River boasts a complex terrain that renders it highly susceptible to geological disasters within China. Thousands of potential geological hazards have been identified in this region, predominantly landslides and debris flows [31]. Despite this, the valley accommodates at least five large hydropower stations. The construction of dams and reservoir impoundment activities have exacerbated ecological issues such as soil erosion, an increased frequency of landslides, the inundation of riparian vegetation, and heightened population density around reservoir areas [31]. Both naturally occurring and human-induced geological threats pose risks of extensive habitat destruction, potentially impacting the survival of *Diploderma daduense* sp. nov.

Despite the array of threats confronting *Diploderma daduense* sp. nov., its current non-endangered status might stem from its extensive distribution range and the prevalence of numerous tributaries within its habitat. However, it remains crucial to diligently monitor the population dynamics of this species to ensure its continued conservation and protection.

## 5. Conclusions

The discovery and formal description of *Diploderma daduense* sp. nov. significantly contributes to our understanding of the diverse genus *Diploderma* (Squamata, Agamidae), now comprising 47 species. Found in the lower Dadu River Valley, Sichuan Province, Western China, this novel species showcases a unique combination of morphological attributes and distinct genetic divergence. Its characterization enhances the knowledge of *Diploderma* species, particularly regarding detailed distribution patterns, morphological variation, and genetic diversity, addressing the paucity of such data for many existing members of the genus. *D. daduense* sp. nov. underscores the critical role of the Hengduan Mountains in speciation processes and provides valuable insights into the evolutionary relationships between the middle and upper reaches of the Yangtze River, as well as the geological history of the Dadu River Valley. This groundbreaking finding sets a solid foundation for future ecological, evolutionary, and conservation research within the *Diploderma* genus and associated disciplines.

**Other examined specimens.**

*Diploderma* sp.1: YBU−20788, YBU−20843, YBU−20801, YBU−20821, YBU−20835 (adult males), YBU−20791, YBU−20873, YBU−20295, YBU−20868, and YBU−20230 (adult females) from Leibo County, Sichuan, China.

*Diploderma* sp.2: YBU−22032 (adult male) from Yibin City; YBU−22035 (adult male) from Naguang, Yibin; YBU−21274 (adult male), YBU−YB1(adult male), YBU−22300, YBU−21273, YBU−23156, YBU−22015, 21029, and YBU−20224 (adult females) from Mt. Qixing, Yibin, Sichuan, China.

## Figures and Tables

**Table 1:** GenBank accession numbers for the sequences used in this study.

	Species	Voucher	Locality	Accession
1	*Diploderma daduense* sp. nov.	CIB−CB2021227	Hanyuan, Sichuan, China	PP539949
		CIB−CB2021228	Hanyuan, Sichuan, China	PP539950
		CIB−CB2021229	Hanyuan, Sichuan, China	PP539951
		CIB−CB2021230	Hanyuan, Sichuan, China	PP539952
		CIB−CB2021237	Jinkouhe, Sichuan, China	PP539953
		CIB−CB2021238	Jinkouhe, Sichuan, China	PP539954
		CIB−CB2021239	Jinkouhe, Sichuan, China	PP539955
		CIB−CB2021240	Jinkouhe, Sichuan, China	PP539956
		CIB−CB2021241	Ebian, Sichuan, China	PP539957
		CIB−CB2021242	Ebian, Sichuan, China	PP539958
		CIB−CB2021246	Shimian, Sichuan, China	PP539959
		CIB−CB2021247	Shimian, Sichuan, China	PP539960
		CIB−CB2021248	Shimian, Sichuan, China	PP539961
		CIB−CB2021249	Shimian, Sichuan, China	PP539962
		CB2021252	Jinkouhe, Sichuan, China	PP539963
		CIB−CB2021253	Shimian, Sichuan, China	PP539964
		CIB−CB2021268	Emeishan, Sichuan, China	PP539965
		CIB−201806029	Jinkouhe, Sichuan, China	PP539941
		CIB−201806030	Jinkouhe, Sichuan, China	PP539942
		CIB−201806031	Jinkouhe, Sichuan, China	PP539943
		CIB−201806033	Jinkouhe, Sichuan, China	PP539944
		CIB−201806034	Jinkouhe, Sichuan, China	PP539945
		CIB−201806036	Jinkouhe, Sichuan, China	PP539946
		CIB−2023SCGZ0302	Jiulong, Sichuan, China	PP539947
		CIB−2023SCGZ0304	Jiulong, Sichuan, China	PP539948
2	*D. splendidum*	CIB−CB20200501	Jiangyou, Sichuan, China	PP539966
		YBU23124(HB1b)	Yichang, Hubei, China	PP539967
		YBU23125(HB2b)	Yichang, Hubei, China	PP539968
		YBU−HB3b	Yichang, Hubei, China	PP539969
		YBU−HB4b	Yichang, Hubei, China	PP539970
		CIB−CB2022Y12	Yichang, Hubei, China	PP539971
		CB2022Y13	Yichang, Hubei, China	PP539972
		CIB−CB2022Y14	Yichang, Hubei, China	PP539973
		CIB−CB2022Y15	Yichang, Hubei, China	PP539974
		CIB−CB2022Y16	Yichang, Hubei, China	PP539975
		CIB−CB2022Y17	Yichang, Hubei, China	PP539976
		CB2022Y18	Yichang, Hubei, China	PP539977
		CIB−CB2022Y26	Yichang, Hubei, China	PP539978
		CIB−CB2022Y29	Yichang, Hubei, China	PP539979
		CIB−CB2022Y30	Yichang, Hubei, China	PP539980
		CIB−CB2022Y31	Yichang, Hubei, China	PP539981
		LFR2023030	Fuzhou, Fujian, China	OR759420
		LFR2023031	Fuzhou, Fujian, China	OR759421
		KIZ015973	Yichang, Hubei, China	MK001418
		LSUMZ81212	Unknown	AF288230
3	sp.1	GP8213YB	Yibin, Sichuan, China	PP539984
		GP8214YB	Yibin, Sichuan, China	PP539985
4	sp.2	GP6871LB	Leibo, Sichuan, China	PP539982
		GP6872LB	Leibo, Sichuan, China	PP539983
5	*D. angustelinea*	KIZ029704	Muli, Sichuan, China	MT577930
		KIZ029705	Muli, Sichuan, China	MT577924
6	*D. aorun*	KIZ032733	Benzilan, Yunnan, China	MT577938
		KIZ032734	Benzilan, Yunnan, China	MT577939
7	*D. batangense*	KIZ09404	Zhubalong, Xizang, China	MK001412
		KIZ019276	Batang, Sichuan, China	MK001413
8	*D. brevicauda*	KIZ044305	Lijiang, Yunnan, China	MW506021
		KIZ044306	Lijiang, Yunnan, China	MW506022
9	*D. bowoense*	KIZ044757	Muli, Sichuan, China	MW506020
		KIZ044758	Muli, Sichuan, China	MW506019
10	*D. brevipes*	NMNS19607	Taiwan, China	MK001429
		NMNS19608	Taiwan, China	MK001430
11	*D. chapaense*	KIZ034923	Lvchun, Yunnan, China	MG214263
		ZMMUNAP−01911	Chapa, Vietnam	MG214262
12	*D. danbaense*	KIZ2022048	Danba, Sichuan, China	OQ378180
		KIZ2022049	Danba, Sichuan, China	OQ378181
13	*D. daochengense*	20210905	Muli, Sichuan, China	OP595620
		DC001	Daocheng, Sichuan, China	OP595621
14	*D. donglangense*	KIZ2022057	Muli, Sichuan, China	OQ378185
		KIZ2022058	Muli, Sichuan, China	OQ378186
15	*D. drukdaypo*	KIZ027627	Jinduo, Xizang, China	MT577950
		KIZ027628	Zhuka, Xizang, China	MT577952
16	*D. dymondi*	KIZ040639	Dongchuan, Yunnan, China	MK001422
		KIZ040640	Dongchuan, Yunnan, China	MK001423
17	*D. fasciatum*	SYS r002175	Wuming, Guangxi, China	OM055809
		KIZ040192	Daweishan, Yunnan, China	OM055800
18	*D. flaviceps*	KIZ01851	Luding, Sichuan, China	MK001416
		KIZ01852	Luding, Sichuan, China	MK001417
19	*D. flavilabre*	KIZ032692	Baiyu, Sichuan, China	MT577916
		KIZ032694	Baiyu, Sichuan, China	MT577917
20	*D. formosgulae*	KIZ044420	Deqin, Yunnan, China	MW506024
		KIZ044421	Deqin, Yunnan, China	MW506025
21	*D. iadinum*	KIZ027697	Yunling, Yunnan, China	MT577956
		KIZ027702	Yunling, Yunnan, China	MT577957
22	*D. jiulongense*	KIZ2022086	Jiulong, Sichuan, China	OQ378190
		KIZ2022087	Jiulong, Sichuan, China	OQ378191
23	*D. kangdingense*	20210916	Kangding, Sichuan, China	OP595625
		20210917	Kangding, Sichuan, China	OP595626
24	*D. laeviventre*	KIZ014037	Basu, Xizang, China	MK001407
		KIZ027691	Basu, Xizang, China	MT577892
		KIZ027692	Basu, Xizang, China	MT577893
25	*D. limingense*	KIZ2022014	Yulong, Yunnan, China	OP428782
		KIZ2022015	Yulong, Yunnan, China	OP428783
		KIZ2022017	Yulong, Yunnan, China	OP428784
26	*D. luei*	NMNS19604	Taiwan, China	MK001433
		NMNS19605	Taiwan, China	MK001434
27	*D. makii*	NMNS19609	Taiwan, China	MK001431
		NMNH19610	Taiwan, China	MK001432
28	*D. menghaiense*	KIZ L0030	Menghai, Yunnan, China	MT598655
		KIZ L0031	Menghai, Yunnan, China	MT598656
29	*D. micangshanense*	KIZ032801	Shiyan, Hubei, China	MK578665
		KIZ023231	Xixia, Henan, China	MK578664
30	*D. panchi*	KIZ032715	Yajiang, Sichuan, China	MT577946
		KIZ032716	Yajiang, Sichuan, China	MT577944
31	*D. panlong*	KIZ040137	Miansha, Sichuan, China	MT577906
		KIZ040138	Miansha, Sichuan, China	MT577907
32	*D. polygonatum*	NMNS19598	Taiwan, China	MK001427
		NMNS19599	Taiwan, China	MK001428
33	*D. qilin*	KIZ028332	Deqin, Yunnan, China	MT577941
		KIZ028333	Deqin, Yunnan, China	MT577942
34	*D. shuoquense*	KIZ2022004	Xiangcheng, Sichuan, China	OP428773
		KIZ2022005	Xiangcheng, Sichuan, China	OP428774
35	*D. slowinskii*	CAS214906	Gongshan, Yunnan, China	MK001405
		CAS214954	Gongshan, Yunnan, China	MK001406
		KIZ027543	Gongshan, Yunnan, China	MT577910
36	*D. swild*	KIZ034914	Panzhihua, Sichuan, China	MN266299
		KIZ034894	Panzhihua, Sichuan, China	MN266300
37	*D. swinhonis*	NMNS19592	Taiwan, China	MK001419
		NMNS19593	Taiwan, China	MK001420
38	*D. tachengense*	KIZ2022028	Weixi, Yunnan, China	OQ378195
		KIZ2022027	Weixi, Yunnan, China	OQ378196
39	*D. varcoae*	WK−JK 011	Yuxi, Yunnan, China	MT577903
		KIZ026132	Mengzi, Yunnan, China	MK001421
40	*D. vela*	KIZ019299	Quzika, Xizang, China	MK001414
		KIZ034925	Quzika, Xizang, China	MK001415
41	*D. xinlongense*	20210907	Xinlong, Sichuan, China	OP595613
		20210908	Xinlong, Sichuan, China	OP595614
42	*D. yangi*	SWFU005410	Chayu, Xizang, China	OL449603
		SWFU005412	Chayu, Xizang, China	OL449604
		SWFU005414	Chayu, Xizang, China	OL449605
43	*D. yongshengense*	KIZ2022009	Yongsheng, Yunnan, China	OP428777
		KIZ2022008	Yongsheng, Yunnan, China	OP428778
44	*D. yulongense*	KIZ028291	Hutiaoxia, Yunnan, China	MT577921
		KIZ028292	Hutiaoxia, Yunnan, China	MT577922
45	*D. yunnanense*	CAS242271	Baoshan, Yunnan, China	MK001408
		KIZ040193	Yingjiang, Yunnan, China	MK578658
46	*D. zhaoermii*	KIZ019564	Wenchuan, Sichuan, China	MK001425
		KIZ019565	Wenchuan, Sichuan, China	MK001426
47	*Pseudocalotes brevipes*	MVZ224106	Vinh Phuc, Vietnam	AF128502
48	*Bronchocela cristatella*	RMB8883	Unknown	KR053114
49	*Laodracon carsticola*	NUOL.R.2022.01	Khammuone, Laos	OR544068

**Table 2:** Morphometric comparison between *Diploderma daduense* sp. nov, sp.1, sp.2, and *D. splendidum*. Morphometric measurements are in the unit of mm. For measurement and count methods and abbreviations, see Section 2.

	*Diploderma daduense* sp. nov		*D. splendidum* (Topotypes)		sp.1		sp.2	
	M22	F11	M6	F5	M4	F6	M5	F5
SVL	74.7–95.0 (86.5)	52.6–80.2 (70.5)	69.7–83.3 (77.1)	63.1–78.2 (70.1)	63.7–94.3 (79.2)	64.6–78.9 (71.0)	77.6–92.2 (84.4)	63.7–72.4 (68.6)
TAL	157.3–245.2 (202.2)	120.0–179.1 (156.2)	164.4–206.8 (190.2)	95.0–180.2 (148.8)	168.4–221.4 (201.1)	162.2–201.2 (177.7)	184.3–205.9 (195.8)	149.9–173.6 (158.9)
HW	14.8–23.6 (20.1)	9.9–13.8 (12.7)	12.7–20.0 (15.5)	10.5–13.7 (12.5)	13.4–21.0 (17.1)	13.2–15.5 (13.9)	15.6–23.6 (19.8)	12.3–14.7 (13.7)
HL	24.0–32.6 (28.6)	15.3–22.9 (19.7)	19.9–27.5 (23.8)	19.6–22.3 (21.0)	20.6–30.3 (26.0)	20.6–22.7 (21.5)	20.0–33.2 (26.9)	20.2–22.2 (21.3)
HD	11.1–16.9 (14.1)	7.8–10.9 (9.7)	9.4–11.6 (10.7)	8.4–10.2 (9.2)	9.7–13.8 (11.8)	9.2–10.4 (9.9)	11.5–16.2 (13.6)	9.2–10.6 (10.1)
SEL	9.6–14.2 (11.3)	6.6–9.1 (8.0)	8.4–11.3 (9.4)	7.9–10.6 (9.1)	9.4–12.4 (11.3)	9.1–9.5 (9.2)	11.6–13.6 (12.3)	9.0–10.2 (9.8)
TNC	1.2–2.9 (1.9)	0.5–1.4 (1.0)	1.0–1.9 (1.2)	0.6–1.1 (0.9)	−	−	−	−
FLL	35.4–44.4 (39.3)	24.0–34.5 (30.5)	34.8–43.9 (38.8)	31.6–37.4 (34.2)	28.3–39.6 (35.6)	26.4–34.9 (31.6)	33.6–40.1 (36.6)	28.5–31.8 (30.4)
HLL	57.1–72.0 (64.3)	34.8–56.8 (52.0)	57.1–73.0 (63.7)	51.4–60.4 (55.1)	50.1–66.3 (61.5)	46.6–59.2 (53.1)	53.6–71.7 (62.0)	48.8–53.5 (51.8)
T4L	13.6–18.3 (15.9)	9.6–15.4 (13.1)	15.0–18.6 (16.6)	13.6–15.2 (14.3)	11.9–16.4 (14.4)	10.7–15.4 (13.3)	13.0–16.8 (15.4)	10.5–13.1 (11.9)
TRL	35.5–49.0 (40.9)	23.4–40.6 (34.4)	32.0–41.1 (36.3)	28.7–39.0 (33.0)	27.7–44.2 (34.0)	29.3–39.7 (34.1)	35.4–42.7 (38.3)	28.5–35.4 (31.7)
TAL/SVL	2.04–2.62 (2.34)	1.89–2.42 (2.22)	2.26–2.70 (2.47)	1.32–2.67 (2.14)	2.35–2.64 (2.55)	2.32–2.63 (2.50)	2.22–2.48 (2.32)	2.14–2.46 (2.32)
HW/HL	0.61–0.79 (0.80)	0.60–0.67 (0.64)	0.59–0.73 (0.65)	0.53–0.65 (0.59)	0.60–0.69 (0.65)	0.61–0.68 (0.64)	0.63–1.06 (0.75)	0.61–0.68 (0.64)
HD/HL	0.42–0.60 (0.49)	0.46–0.54 (0.49)	0.42–0.47 (0.45)	0.42–0.47 (0.44)	0.44–0.47 (0.45)	0.42–0.50 (0.46)	0.46–0.71 (0.52)	0.46–0.49 (0.47)
HD/HW	0.63–0.90 (0.70)	0.72–0.82 (0.76)	0.58–0.77 (0.70)	0.65–0.88 (0.74)	0.65–0.76 (0.70)	0.67–0.78 (0.72)	0.67–0.73 (0.69)	0.71–0.78 (0.73)
SEL/HL	0.36–0.44 (0.39)	0.36–0.47 (0.41)	0.36–0.42 (0.40)	0.40–0.51 (0.43)	0.41–0.46 (0.44)	0.41–0.44 (0.43)	0.41–0.63 (0.47)	0.45–0.47 (0.46)
FLL/SVL	0.41–0.49 (0.45)	0.38–0.49 (0.44)	0.47–0.54 (0.50)	0.45–0.52 (0.49)	0.42–0.51 (0.45)	0.36–0.50 (0.45)	0.41–0.45 (0.43)	0.42–0.47 (0.44)
HLL/SVL	0.67–0.81 (0.74)	0.66–0.81 (0.75)	0.78–0.89 (0.83)	0.77–0.81 (0.79)	0.70–0.87 (0.78)	0.64–0.87 (0.75)	0.67–0.78 (0.73)	0.73–0.83 (0.76)
TRL/SVL	0.43–0.52 (0.47)	0.45–0.54 (0.49)	0.41–0.53 (0.47)	0.45–0.50 (0.47)	0.43–0.47 (0.45)	0.45–0.51 (0.48)	0.41–0.49 (0.45)	0.43–0.49 (0.46)
HL/SVL	0.28–0.36 (0.33)	0.27–0.30 (0.29)	0.28–0.33 (0.31)	0.28–0.31 (0.30)	0.32–0.34 (0.33)	0.29–0.32 (0.30)	0.23–0.36 (0.32)	0.30–0.32 (0.31)
TNC/HL	0.04–0.09 (0.06)	0.03–0.07 (0.05)	0.04–0.07 (0.05)	0.03–0.06 (0.04)	−	−	−	−
MD	45–55 (50.8)	47–57 (50.7)	46–55 (49.8)	40–62 (51.9)	−	−	−	−
GU	28–36 (33.0)	27–36 (30.6)	27–33 (30.0)	24–36 (31.7)	−	−	−	−
VN	51–68 (59.0)	55–63 (59.1)	58–64 (60.7)	51–68 (59.6)	−	−	−	−
F4S	18–23 (19.4)	17–23 (19.2)	19–22 (20.5)	18–23 (19.9)	−	−	−	−
T4S	21–31 (25.5)	21–28 (24.8)	24–28 (26.0)	21–31 (26.0)	−	−	−	−

## Data Availability

The data presented in this study are available on request from the corresponding author.

## Supplementary Materials

The following supporting information can be downloaded at https://www.mdpi.com/article/10.3390/ani14091344/s1, Table S1: Morphological data of the type series of *Diploderma daduense* sp. nov and *D. splendidum*. Morphometric measurements are in the unit of mm. For measurement and count methods and abbreviations, see the Materials and Methods, Table S2: Uncorrected genetic pairwise distances (*p*-distances) (%) between *Diploderma* species in this study based on the mitochondrial *ND2* gene sequences, Table S3: Summary statistics of PCA of males’ morphometric characteristics in selected members, Table S4: Summary statistics of PCA of females’ morphometric characteristics in selected members.
